# The experience of Italian student athletes enrolled in a dual career university program: the challenges of employability

**DOI:** 10.3389/fspor.2024.1515634

**Published:** 2025-01-09

**Authors:** Mattia Belluzzi, Alessia Ferraboli, Caterina Gozzoli, Chiara D’Angelo

**Affiliations:** Department of Psychology, Catholic University of the Sacred Heart, Milan, Italy

**Keywords:** graduate employability, athletes, dual career, career transitions, student-athletes

## Abstract

**Introduction:**

In the so-called “new career era,” career transitions and employability have attracted growing attention. For elite athletes, the Dual Career experiences may be regarded as a protective factor during various career transitions, with the end-of-career transition being closely linked to employability. This contribution aims to explore the dual career paths of athletes through the lens of graduate employability, as perceived by the individuals themselves. Thus, employability can be considered as an important resource for athletes during the many transitions that characterize their sports careers.

**Methodology:**

Study participants included 21 student-athletes participating in the first four editions of the Dual Career program at an Italian university. Using a phenomenological-interpretive approach, semi-structured interviews and focus groups were conducted with participants. A top-down and bottom-up content analysis was conducted using Nvivo software for qualitative analysis.

**Results:**

The data derived from interviews and focus groups are presented with reference to four dimensions—Human Capital, Social Capital, Individual Behaviors, and Individual Attributes—that, according to Clarke's integrated model, impact perceived employability in graduate students, including student-athletes. The results highlight traits shared with classic university students while, at the same time, emphasizing peculiar sub-dimensions.

**Discussion:**

This study integrates the constructs of employability and career transitions in the target group of university athletes. Through the Clarke's model, which offers a complex and multidimensional definition of employability, this research explores why the dual career pathway represents a resource for athletes during transitions. The results can serve as a first step in developing good practices for supporting and guiding student-athletes enrolled in Dual Career programs, by addressing aspects recognized in the scientific literature as practical implications for supporting employability development.

## 1 Introduction

Contemporary labor markets are undergoing significant economic changes (e.g., globalization) as well as technological changes (e.g., the rise of artificial intelligence), leading individuals to increasingly change jobs, employers, and/or professions multiple times throughout their careers (1, 2). In the so-called “new career era,” career transitions and employability have attracted growing attention from scholars in the fields of professional and organizational psychology (3). While contemporary career literature implicitly or explicitly assumes a strong connection between the two concepts, there has been limited theoretical or practical exchange between the two fields [(4), p. 799]. In the contemporary career context, it is generally acknowledged that there is no ideal career path characterized by a set of predictable transitions that all workers go through at specific points in their lives (5). Career transitions, recurring throughout the lifespan (6), thus instead refer to every move from one position to another within social spaces (7). The notion of “one life, one career” (8) is shifting towards much more complex and idiosyncratic career models, making the individual the central actor (5, 9).

One population particularly affected by career transition dynamics is that of elite athletes—that is, sportsmen and sportswomen competing at the national or international level. During their sports careers, which are characterized by brevity compared to other professions, athletes face numerous transitions. Sports transitions can be normative (i.e., predictable, such as the Junior to Senior transition), non-normative (i.e., unpredictable, such as career-ending injuries) (10), and quasi-normative (i.e., predictable transitions for a group of athletes, such as cultural transitions (11);. The Holistic Athletic Career Model (12) explores the development of athletes through five different levels—athletic, psychological, psychosocial, academic/vocational and financial levels—which are characterized by various transitions faced by the same individual.

The inclusion of an academic dimension in the study of athletic careers makes it possible to contextualize both athletic and academic careers (13). In recent years, increasing attention has been paid in the sports context to the concept of Dual Career (DC), understood as the opportunity for elite athletes to successfully initiate, develop, and terminate both a sports and educational or work-related path (14, 15). Dual career athletes manage the demands of education, training, and future employment alongside their athletic careers, all within the larger frameworks of sports management and education (16, 17). DC is seen as a challenge for athletes and their environment (18), but it can also be considered as a protective factor for identity definition during critical transition moments, especially at the end of their careers (19, 20). Among the various career transitions, one of the most delicate for athletes is retirement. Sooner or later, every elite athlete faces retirement, and athletes' retirement is a multifaceted, complex, and subjective phenomenon (21, 22). Researchers have examined various factors (e.g., athletic identity, voluntary career termination) influencing the quality of athletes' transitions (20, 23, 24). In this sense, research has highlighted the importance of transition in athletes' lives and the challenges they face in their post-athletic life (25). These challenges include uncertainties related to employment and potential lifestyle changes; feelings of vulnerability in managing changes in their expectations, goals, and plans; anxiety about the unknown (26); and barriers to reaching a satisfactory job position (27). In this context, DC has the potential to support athletes' readiness for a working life after sports retirement, as the majority of them must rely on another professional occupation to sustain themselves after sport (28).

The theme of end-of-career transition is closely related to the construct of employability, which has evolved over the years from a strictly individual perspective determined by the human capital needed to enter a specific professional field and have a linear career (5) into a “multidimensional, lifelong, and life-wide” phenomenon (29, 30). The personal perception of opportunities in the internal and external job market is increasingly emphasized, as well as how this perception is influenced by individual and contextual factors and is related to career decisions and subsequent outcomes (31). A target group that is strongly interested in the construct of employability, due to the urgency of employment, is that of graduates. Thus, Higher Education Institutions are key actors in the DC reality concerning employability development (28).

Over the years, two consolidated models of occupational capital have been established and verified to prepare university students for the transition to the job market through empirical evidence. Tomlinson's model (32) defines graduate capital as an accumulation of five forms of capital: human, social, cultural, identity and psychological. Clarke (33) defined an integrated model conceptualizing employability “*as comprising the human capital, social capital, and individual behaviours and attributes that underpin an individual's perceived employability, in a labour market context, and that, in combination, influence employment outcomes*” [(33), p. 1931]. Human Capital is a core component because the development of skills and competences is considered significant for tertiary educational paths, being associated with occupational expertise. Combined with Social Capital and its components—university ranking, network and social class—this form of capital enhances graduate employability. Individual attributes and behaviors are acknowledged as essential to achieving career success. Finally, the current labor market shapes both perceived employability and graduate employability.

For elite athletes, the issue of employability is a continuous concern throughout their sports careers, primarily due to widespread contractual instability in the sports field, difficulties in finding sustainable work compatible with their sports career, the complexity of seeking alternative employment following injuries, and the challenges female athletes face in continuing their careers after pregnancy (34, 35). One of the primary challenges in the European discourse on Dual Career is to promote “sport and education within the business community in Europe” [(18), p. 86]. The construct of employability can thus be thought of as a bridge between the sports and educational/work-related paths at various points in the sports career, including: when they are still active in elite sports (active phase), when they are planning to retire or have already retired and are not (yet) employed in their post-sports career (retirement phase), and when they are employed in their post-sports career (new career phase) (36).

In summary, student-athletes face numerous challenges and transitions at different levels during their lives. From a holistic perspective, academic and sport dimensions are considered integrated and mutually influential. Furthermore, given the brevity of athletic careers, athletes face the need to integrate into the labor market upon retirement. As highlighted by Stambulova and Wylleman (18), graduation from university is considered as a transition in which athletes play an active role in shaping their career paths. We thus hypothesize that DC may serve as a supportive factor in the employability development process for this specific target group. As the target group are also students, the construct of graduate employability has been chosen as a reference, particularly according to Clarke's (33) model.

The present study addresses the challenge posed by Stambulova and Wylleman (18) regarding athletes' employability challenges, linking it to dual career in sport and education. This contribution aims to explore the dual career experiences of athletes through the lens of graduate employability, as perceived by the individuals themselves. Hence, two research questions will guide this study:

(1) Which dimensions of DC are connected with graduate employability?
(2) How can the DC experience serve as a resource for athletes during the many transitions that characterize their sports careers?

## 2 Methodology

### 2.1 Participants

A purposive sampling technique was applied to involve 21 student-athlete participants (9 male and 14 female) from the first four editions of the Dual Career Program at an Italian University. Having participated in the Program for at least one year was set as the only inclusion criterion.

Participants practiced 17 various sports, both individual (e.g., motocross, karate, water skiing, wakeboarding, etc.) and team-based (e.g., volleyball, rugby, water polo, etc.).

Sixteen of the student-athletes were studying for a Bachelor's degree, four for a Master's degree, and one for a Single-cycle Master's Degree related to 6 different Faculties (e.g., Economics, Educational Sciences, Political and Social Sciences, etc.) and 11 diverse academic programs (e.g., Economics and Management, Sports and Exercise Sciences, etc.).

### 2.2 Procedure and instruments

Based on a phenomenological-interpretive approach (37), seven semi-structured interviews and three focus groups were conducted with the participants. Each focus group involved from three to six student-athletes.

The aim was to explore participants' perception of the DC Program attended and how it could develop their employability and serve as a resource for future career transitions. The interview and focus group guides were identical, including questions about their sports and academic careers and the intersections of the two paths, as well as the skills, learning, and awareness developed within them. The interviews and focus groups were conducted from 2022 to 2023 and lasted between 25 and 50 min.

The study's objectives were explained to all participants, and they were assured of the confidentiality of their responses, the anonymity of data processing, and their right to withdraw at any time. All provided their informed consent, and various tools (i.e., video calls) were used to overcome some scheduling difficulties due to the sports and academic commitments of the student-athletes. The interviews were audio-recorded, transcribed verbatim, and subjected to content analysis. The study was approved by the Ethics Committee of the Department of Psychology of the authors' university.

### 2.3 Data analysis

The analysis was conducted by the first author using NVivo software for qualitative analysis. A coding framework based on Clarke's integrated model of perceived employability in university students (33) was created for this purpose. Relevant themes selected were (a) human capital, (b) social capital, (c) individual behaviors, and (d) individual attributes. Following an initial familiarization with the data, they were coded by applying the four identified categories.

Subsequently, according to the standard IPA technique (37), transcriptions were analyzed using a bottom-up approach, remaining open to codes emerging from the data. Initial coding was performed, identifying significant aspects in each transcription, and codes were later aggregated in themes. The analysis was then verified and revised multiple times by all authors until a triangular consensus was reached (38, 39).

## 3 Results

Data derived from the analysis of interviews and focus groups are presented with reference to four dimensions—Human Capital, Social Capital, Individual Behaviors, and Individual Attributes—that, according to Clarke's integrated model (33), impact perceived employability in graduate students, including student-athletes. We consider it appropriate to use these dimensions because they refer to a subjective dimension of employability which we believe is consistent with the target group of student-athletes, the DC program, and the methodology used.

### 3.1 Human capital

Despite the ongoing debate regarding the transferability of soft skills, their development remains a marking peculiarity of graduates (33).

In the present study, the participants defined the dimension of competences included in Clarke's model (33), highlighting the skills and abilities that a Dual Career path allows them to develop. Foremost among these is organization, understood as the ability to balance two very demanding paths while thinking ahead to future competitions and exams:

“The ability to organize oneself not only in the short term, during the week, but also in the long term, thinking about the competitions I already know I will participate in, as well as the exams and assessments that I have in my study plan.” (M, swimming)

Coping skills and problem-solving also emerge in interviews as competences that can be mutually reinforcing across the two paths, helping student-athletes to face challenging situations:

“The ability to face an exam as you have learned to face a competition, a match.. you learn how to handle an exam or a degree or other important commitments..” (M, canoeing)

“Being able to solve problems quickly, as being exposed to various situations, they can solve a problem quite quickly.” (F, sport dance)

Others underlined the development of stress management competence, understood as

“The ability to manage various pressures from all points of view, from sports, parents, coaches, university, friends.” (M, sport dance)

Participants also considered teamwork as a fundamental competence, because participating in project gives them the feeling of belonging to a team that can support them and to which they can turn for help in achieving their goals.

**Figure 1:**
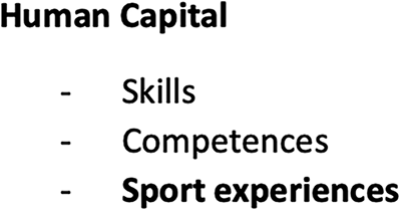
Dimensions of human capital.

The sports experience of student-athletes integrates the work experience of internships and traineeships in Clarke's model (see Figure 1) and enhances the development of specific competences and skills:

“If we think about a student who only studies, in practice, he devotes himself full-time to his studies. On the other hand, a professional student-athlete performs two full-time jobs in one day and would like to do both well.” (M, water skiing)

### 3.2 Social capital

The equations should be inserted in editable format from the equation editor.

University reputation and the possibility of activating networks are relevant elements concerning student-athletes' perception of their employability. The participants’ university offers access to excellent services and contacts with companies, fostering internship and future employment opportunities.

It could also be suggested that unique features of the DC Program can further enhance social capital. These including meeting sessions with other student-athletes are planned and the distinctive DC experiences of student-athletes that are shared on the university website.

Additionally, and specific to the target of student-athletes, the support received from tutors of the DC Program emerges as a significant dimension of social capital. Tutors not only offer organizational assistance but also provide emotional and psychological support:

“I thought I could do everything without help, but the support I received from other people during this path.. made me realize that I am not alone.” (M, fencing)

**Figure 2:**
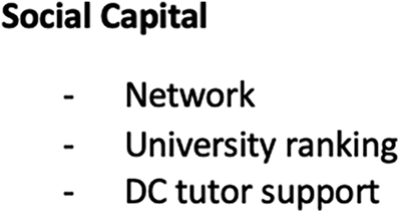
Dimensions of social capital.

Social class dimension did not emerge as a significant theme in this study (see Figure 2).

### 3.3 Individual behaviors

**Figure 3:**
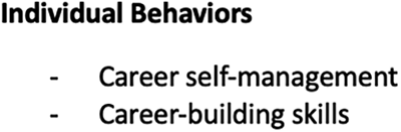
Dimensions of individual behaviours.

The sub-dimensions of career self-management and career-building skills find ample representation in the words of student-athletes (see Figure 3). Regarding the former, a valuable element in the path is represented by the support to student-athletes in developing self-awareness and emotional intelligence, and in designing their future

“I have become more aware of what I really wanted and how to achieve it.. in fact, I decided to change coach because it had been a long time since I felt comfortable with the old one.” (F, athletics)

“It’s like putting myself in someone else’s shoes who is in my situation.. this emotional intelligence that I would have had with someone else, I had it with myself.. because I had it with myself, I was able to develop it to have it with my friends or other people.. I must say that I started to look at things from other emotional perspectives.” (M, rugby)

The sub-dimension of career-building skills also emerged as central in the DC path, particularly in terms of seeking help, determination, and resilience:

“Even asking for help may seem obvious but it isn’t, both in university and in sports. You have to accept your limits and understand that if you continue like this, you can also fall..” (F, waterpolo)

“It’s about having hunger, we want to get there, having the determination to achieve a goal.” (F, ski)

“Maybe I’m saying something obvious, but for me, resilience comes to mind in the sense that there are more failures than successes, and it’s the ability to always put oneself on the line, the willingness to do it over and over again.” (M, swimming)

### 3.4 Individual attributes

**Figure 4:**
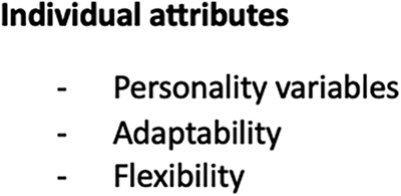
Dimensions of individual attributes.

Personality variables constitute a sub-dimension that recognizes the individual traits of each student-athlete, which impact their self-perception and the path they are facing. Additionally, both adaptability and flexibility are skills that each student-athlete develops, both during their sports career and once they have undertaken the DC (see Figure 4):

“And then there’s flexibility in the sense that my days were very intense and I knew I didn’t have a routine. I am usually rigid and follow a routine, and I have learned to be flexible.” (F, Karate)

“Basically, I changed coaches every 2 years, each has their own method and style, there are the more sympathetic and the less sympathetic ones, you have to adapt to what they ask you for at that moment and try to make your teammates accept it.” (F, rugby)

## 4 Discussion

This paper presents a qualitative study focused on exploring student-athletes' perceptions of their DC experiences through the lens of graduate employability.

Findings highlight that the construct of employability in the target group shares commonalities with “classic” university students but, at the same time, emphasizes peculiar sub-dimensions. Foremost, the support provided to student-athletes, not only from an organizational point of view but also (and especially) psychosocially, emerges as crucial in the process of attributing meaning to both sports and academic experiences.

**Figure 5:**
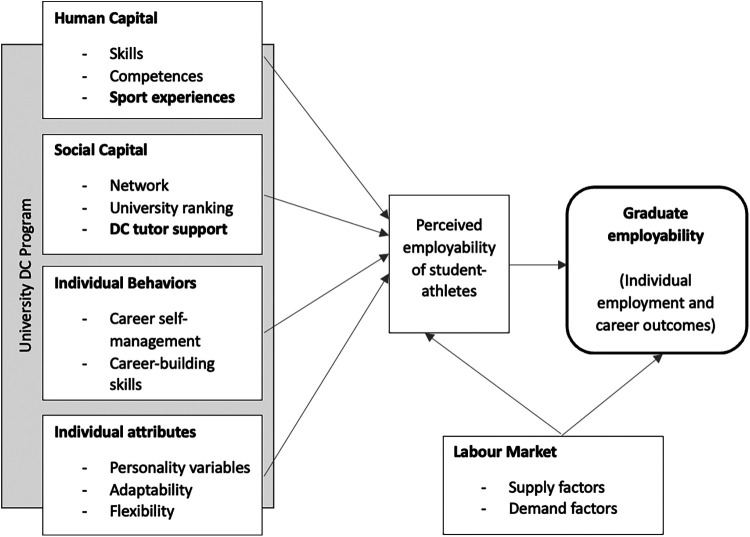
Integrated model of graduate employability for student-athletes (adapted from Clarke's integrated model, 2018).

Within a DC path, the core components of graduate employability summarized in Clarke's model (33) can be enhanced (see Figure 5). The Human Capital of student-athletes is expressed through the development of competences and skills that are both enhanced and used in academic and sports domains. Furthermore, sports experiences are considered similar to the work experiences described in the model. Attending a prestigious university and engaging in diverse networking opportunities beyond sport, combined with the unique relationship with DC tutors, enables student-athletes to cultivate Social Capital. Finally, Individual Attributes and Behaviors of student-athletes require support in developing self-awareness and reflecting on these dimensions.

It is noteworthy that these dimensions are central in preparing the athlete for typical career transitions, both athletic and non-athletic. Consequently, employability could be understood in a broader sense than the early conceptions of “possession” (40), as a dimension that facilitates effective transition. In light of the fact that athletes' career development is characterized by diverse transitions across different developmental levels (12), student-athletes significantly emphasized the importance of organizational guidance (e.g., planning academic and sports commitments) and psychological support provided through the DC Program for successful transitions in both athletic (i.e., changing teams and coaches) and non-athletic (i.e., from high school to university) domains. Planning for retirement, developing multiple identities, and preserving a broad network of social relationships in both sporting and non-sporting environments are essential processes that guide successful transitions within and outside sport (41). Moreover, student-athletes defined various competences (i.e., organization, coping skills, problem-solving, stress management, and teamwork) developed through DC experiences that could be considered as resources during career transitions.

Conceiving sports and athletic careers as places and spaces for personal and professional growth, DC Programs are in line with professional development interventions conducted outside the curriculum (42). The role of DC tutors in guiding student-athletes in reflecting on themselves—developing strong self-awareness and understanding their strengths, areas for improvement, and values, which are conceived not as fixed but as continuously evolving—represents an important element to move in the direction of identity openness.

The construct of identity plays a significant role in the effectiveness of a transition (43), particularly in the case of athletic career transitions. In major cases of athletes engaging in elite competitions, the prevailing dimension is that of athletic identity. Defined as the degree to which an individual feels like and thinks of oneself as an athlete (44), athletic identity is positively associated with athletic performance. It is also one of the main factors affecting athletes' personal and psychological development, subsequent adjustment difficulties following retirement from sports, post-injury emotional distress, and social isolation (45). When individuals approach the end of their athletic career, they tend to proactively reduce the personal importance of their athletic identity and explore neglected, abandoned, or entirely new dimensions of their identity. This creates a conflict in the athlete between *what is* and *what they want* or *what should be*, stimulating them to mobilize resources to cope with the challenges of the process. Moreover, in a population that by definition focuses on short-term goals and the “here and now,” increased planning for the future is an element that impacts perceived employability and prevents some pitfalls of end-of-career transitions. The typical mode of an athlete is to act in an “interventionist” manner on the problem at hand. Being in a DC pathway can help the athlete in building long-term thinking and broader planning by shifting focus to the “there and then.”

If employability capital can be developed through person-centered pedagogical practices (46), problem-based learning (47), and opportunities for personal reflection (48), being in a pathway that works on developing self-awareness and at the same time develops an identity alternative to the athletic identity (e.g., academic or professional) can truly represent a protective factor in transitions. Employability is therefore closely linked to individuals' identity and the transitional moments they go through (49). With this approach, the typical change experienced during transitions will not be understood as mere adaptation and management of new life events but, rather, as a real transformation that concerns one's existence and, consequently, one's identity (49).

The goal of reflexive practice (50) should therefore be to develop a flexible identity construction that adapts well to “liquid modernity” (51) and promotes thinking about a life plan, whether inside or outside of sports. An important element is thus to activate awareness and reflection on how the two paths—academic and athletic—can be intertwined and not just parallel. Parallelism implicitly brings with it the idea of two things that never touch; in contrast, the idea we support within DC pathways is that what develops in one context, if reworked, can constitute a resource for the other. So far, we have only considered dimensions pertaining to the intrapersonal sphere, which includes the constructs of identity, awareness, and long-term thinking. However, a more contextual and systemic perspective should not be overlooked. The literature highlights the interconnected and interdependent nature of different actors operating within a work ecosystem (52). In the Employability Capital Growth Model (ECGM), Donald and colleagues (42) have highlighted how graduate students, educators, career professionals, and employers are the main stakeholders; in the target group of student-athletes, key actors from the sports world should be added, such as coaches, agents, and managers.

To move towards the dimensions highlighted in the results and discussion, a change is needed in the approach characterizing the sports/education and sports/labor world dichotomies. The tendency for these contexts to not communicate and to view each other with suspicion and distrust, in the belief that one takes away space and energy from the other, should be replaced with a more harmonious integration between sports and education. This approach will make it possible to develop athletes with separate but interconnected identities, which can constitute an invaluable resource even in the delicate moment of end-of-career transition.

## 5 Limitations

This study focuses on student-athletes enrolled in the DC Program of a single university, which represents only one of the network of Italian universities committed to promoting DC programs to students. It would be interesting to extend the study to other DC programs, both Italian and European, to explore whether the specific design and development approach of each university impacts dimensions related to perceived employability and, consequently, the typical transitions of the target group. Additionally, the study provides a snapshot of the present moment without longitudinally monitoring the subjects' developments, transitions, and future employment as an outcome of employability. Further research could also develop this temporal dimension by collecting data from the same subjects at multiple points in time.

## 6 Conclusions and practical implications

To the best of the authors' knowledge, there are no studies in the literature to date that integrate the constructs of employability and career transitions in examining the target group of university athletes. These constructs, which can be seen as two sides of the same coin, seem to represent an interesting lens for designing and developing support services for student-athletes and preventing unemployment and difficulties at the end of their sports career and during the transitions they experience. The results of the present study can thus serve as a first step in developing best practices for supporting and guiding student-athletes enrolled in Dual Career programs by addressing aspects recognized in the scientific literature as practical implications for supporting employability development, such as goal-oriented behaviors, taking ownership of one's career, developing networks, engaging in continuous and lifelong learning, and understanding the value of work experience. Thus, the construct of employability, in its broader definition beyond mere possession of skills useful to the labor market, can serve as a common ground for dialogue between the sports, educational, and (in the future) labor worlds. This is true not only at the specific moment of end-of-career transition—when the question of “who am I now” and the activated skills becomes urgent and emergent—but throughout the athlete's whole sports and educational journey, encompassing various but equally important transitions, shaping athletes' perceived employability.

## Data Availability

The raw data supporting the conclusions of this article will be made available by the authors, without undue reservation.
